# Nitrogen Isotope Effects in Urea Metabolism: From Biochemistry to ^15^N Natural Abundance in Cancer

**DOI:** 10.3390/ijms27083462

**Published:** 2026-04-12

**Authors:** Illa Tea, Guillaume Tcherkez

**Affiliations:** 1 Université Lyon 1, CNRS, ISA UMR 5280, 69100 Lyon, France; 2 Division of Plant Sciences, Research School of Biology, The Australian National University, Canberra, ACT 2601, Australia; guillaume.tcherkez@anu.edu.au; 3 Institut de Recherche en Horticulture et Semences, INRAE, Université d’Angers, 49070 Beaucouzé, France

**Keywords:** ^15^N, urea cycle, isotope effects, natural abundance, cancer metabolism

## Abstract

The urea cycle (UC) is usually described as the hepatic metabolic pathway responsible for ammonia detoxification, but its role extends far beyond nitrogen (N) elimination to include cellular biosynthesis and metabolic signalling. In cancer cells, the UC is reconfigured/reorchestrated to support high anabolic demand, often involving the dysregulation of key enzymes such as ASS1, ASL, OTC and CPS1. While these changes support biomass production and stress resistance, they also generate measurable biochemical signatures through kinetic and thermodynamic isotope effects (^14^N/^15^N). In this review, we summarise UC biochemistry and recall key enzymatic mechanisms. Together, these elements provide a mechanistic framework to interpret changes in ^15^N abundance observed in tumour tissues and cells. We discuss how the redirection of N flux toward nucleotide and polyamine synthesis, coupled with partial excretion of ^15^N-depleted urea, may shape the isotopic composition of cancer cells. By integrating molecular oncology with stable isotope analysis, this review highlights the potential of natural isotope abundance as a functional readout of tumour metabolism and supports further investigation of its translational relevance in cancer phenotyping and monitoring.

## 1. Introduction

The urea cycle (UC) is usually described as the hepatic metabolic pathway that generates urea and is responsible for the detoxification of ammonia. Its functions extend far beyond the elimination of nitrogen (N) and are closely linked to cellular biosynthesis and metabolic signalling. Under physiological conditions, proximal (carbamoyl phosphate synthase, CPS1, and ornithine transcarbamoylase, OTC) and distal (argininosuccinate synthase, ASS1, argininosuccinate lyase, ASL, and arginase, ARG) enzymes of the UC convert ammonia into urea while producing arginine, citrulline, and ornithine as intermediates [1]. The UC is at the crossroads of various metabolic pathways, since it connects to (i) the tricarboxylic acid (TCA) cycle via fumarate, (ii) nucleotide biosynthesis through aspartate and carbamoyl phosphate (CP), (iii) polyamine metabolism via ornithine, and (iv) redox metabolism through the aspartate-arginosuccinate shunt, which couples N flux to fumarate production and NADH/NAD^+^ balance [1]. These metabolic interconnections are illustrated by congenital UC-related disorders, where alterations of UC intermediates disrupt various aspects of metabolic homeostasis even in the absence of hyperammonemia [2]. This also suggests that changes in UC activity may have broader metabolic consequences in other pathological contexts, including cancer.

**Figure 1:**
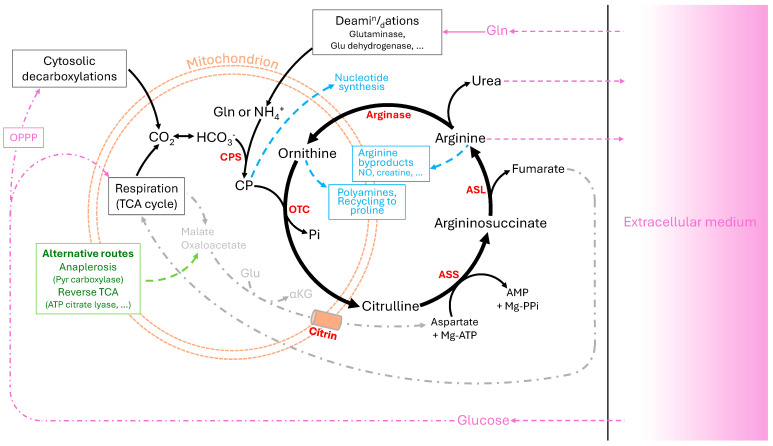
Summary of urea cycle reactions and their interactions with other metabolic pathways. Solid black arrows indicate the main urea cycle reactions; dashed arrows indicate metabolic exchanges or connections with associated pathways. The orange dashed outline indicates the mitochondrion. Key enzymes (and the Asp transporter) are highlighted in red (see the text for details). Metabolite exchange with the extracellular environment is shown with dashed pink arrows. Abbreviations: αKG, 2-oxoglutarate (α-ketoglutarate); CP, carbamoyl phosphate; Gln, glutamine; Glu, glutamate; OPPP, oxidative pentose phosphate pathway; TCA cycle, tricarboxylic acid cycle (Krebs cycle).

Increasing evidence shows that UC enzymes are affected in cancer cells, contributing to anabolic metabolism, redox balance and adaptation to nutritional stress (Figure 1). Tumour cells redirect carbon (C) and N fluxes and thus reprogram metabolic routes to support high anabolic demand associated with intense proliferation. Amongst reprogrammed pathways, UC has emerged as a compelling example of metabolic flexibility in cancer [1]. In fact, many tumours show altered expressions of CPS1, OTC, ASS1, ASL, ARG, Citrin, and amino acid transporters [3], thereby impacting the metabolic partitioning of polyamines and organic acids (Figure 1). Such changes reduce N efflux from the cell and favour N retention for biosynthesis of nitrogenous compounds [1,4]. Aspartate and CP can be redirected to nucleotide synthesis, while arginine and polyamines support cell proliferation [2,5]. This rewiring also generates oncometabolites, such as fumarate, which modify signalling pathways, epigenetic regulation and cellular plasticity [6,7]. It has been argued that ASS1 suppression in several tumour types redirects aspartate towards pyrimidine synthesis, enhancing proliferation, while conferring sensitivity to arginine deprivation [5]. Similar UC alterations have been described across solid tumours and blood malignancies, suggesting that UC can be a target for chemotherapy [8]. Collectively, the enhancement of UC in cancer cells contributes to biomass production, stress resistance, and microenvironment remodelling but also represents a metabolic vulnerability: arginine auxotrophy and aspartate dependency [9].

Interestingly, such a metabolic reorchestration has measurable biochemical consequences on N isotope distribution (i.e., natural abundance of the stable isotope ^15^N compared to that of ^14^N). N-transfer reactions exhibit kinetic isotope effects (^14^*k*/^15^*k*, explained below), which can propagate through metabolic networks and result in detectable signatures in tissues, biological fluids and excreted metabolites [10,11,12,13,14,15,16,17,18]. In the case of cancer, N metabolism as a whole is reshaped, with an increase in glutaminolysis. In principle, changes in UC metabolic flux should lead to an increase in the demand for arginine, dysregulated polyamine synthesis, and a modification of ammonium and urea concentration. As a result, cancer cells may exhibit a specific natural abundance in ^15^N. If true, natural isotope composition could provide a useful functional readout of tumour N fluxes and a rationale for future studies on cancer phenotyping or monitoring.

In this review, we summarise UC biochemistry, ^14^N/^15^N isotope effects and cancer metabolic reprogramming to explain how UC links to the natural abundance of ^15^N in tumours. Section 2 presents principles of isotope effects and enzymatic metabolism in cancer cells and explains why cancer cells provide a unique biochemical context to investigate natural modifications of ^15^N abundance. Section 3 examines enzyme-specific N isotope effects in the UC and thus provides the chemical background to explain the ^14^N/^15^N selectivity of UC. Section 4 embraces the literature showing the natural ^15^N depletion observed in cancer tissues and cells. Section 5 includes perspectives, in particular, for other isotopes, such as ^13^C, ^34^S, and metal isotopes.

## 2. Basics of Nitrogen Isotope Effects and UC Metabolism in Cancer Cells

### 2.1. Nitrogen Isotope Composition, Isotopologues and Isotope Effects

Biological macroelements (C, H, O, N and S) occur naturally in different isotopic forms, with characteristic proportions varying across organs, tissues, metabolites, and even between distinct atomic positions within a molecule. For example, N is present mostly as ^14^N (about 99.63%), while the heavier isotope ^15^N represents about 0.37%. Yet, the relative abundance of ^15^N is not constant, with very small—but easily measurable—differences between dietary and endogenous proteins, tissues, and N-atom positions within amino acids and other nitrogenous metabolites. Such variations arise from isotope effects, whereby enzymatic reactions, transport processes, or phase transitions discriminate between molecules carrying ^14^N or ^15^N (isotopologues, see also below). Understanding and quantifying these effects requires a few key concepts described below.

The abundance of ^15^N is expressed with the ‘isotope composition’ or ‘delta value’. It is computed using the heavy-to-light ratio (^15^N/^14^N) in the sample of interest, relative to an international reference (also called ‘standard’). The isotope composition is usually measured by isotope ratio mass spectrometry (IRMS), denoted as δ^15^N or simply δ, and is expressed in per mil (‰, also sometimes denoted as mUr for milli-Urey). δ can be negative, reflecting ^15^N-depletion with respect to the international standard, while positive δ indicates a ^15^N-enrichment. δ is calculated as follows:

$$\delta (‰)=\left(\frac{R_{\mathrm{sample}}}{R_{\mathrm{standard}}}-1\right)\times 1000$$

where $R_{\mathrm{sample}}$ is the isotope ratio (^15^N/^14^N) in the sample, and $R_{\mathrm{standard}}$ is the isotope ratio of the international reference standard. Since the gas analysed by IRMS is N_2_, the international standard for nitrogen isotopes is atmospheric dinitrogen (abbreviated AIR in isotopic parlance).

Isotopologues are molecules that differ only by the presence of a heavy isotope at one or more positions. For example, dinitrogen (N_2_) has two isotopologues, ^15^N–^14^N and ^14^N–^14^N. On the other hand, isotopomers are structural isomers differing in the position of the isotopic substitution. For example, in glutamine, ^15^N can be located either on the amide nitrogen (γ-N) or on the α-amine nitrogen, giving two isotopomers with the same molecular formula but different N-atom positions of ^15^N. In practice, when nitrogenous metabolites have multiple N-atoms, there is quite a number of isotopologues/isotopomers possibilities, meaning that in nature, such compounds are present as complex isotopic mixtures. For example, in the case of arginine, C_6_H_14_N_4_O_2_, there are 2^4^ = 16 isotopic species when considering N alone, and more when C, H and O isotopes are also taken into account. The isotope composition obtained by IRMS provides an average (whole-molecule ^15^N abundance) but does not provide information on positional ^15^N abundance at each N-atom position. Unlike for natural ^13^C abundance, which can be resolved at the intramolecular level (i.e., so-called ‘site-specific’ analysis) by nuclear magnetic resonance (NMR), there is presently no suitable, routinely implementable technique for site-specific ^15^N analysis. That said, methodological advances are beginning to narrow this gap. Compound-specific δ^15^N analysis of amino acids by gas chromatography–combustion–IRMS (GC-C-IRMS) continues to improve through optimised derivatization and identification workflows, while recent quantitative ^15^N NMR and intramolecular analytical approaches suggest that position-specific ^15^N analysis at natural abundance may become more accessible in the future. High-resolution mass spectrometry may also offer complementary opportunities for low-abundance samples. Although these methods are not yet routinely implementable for UC metabolites in biomedical studies, they open promising prospects for resolving N-atom-specific isotope effects in molecules such as arginine, ornithine or glutamine. In the case of UC biochemistry, this remains an important limitation because enzymatic isotope effects impact specific N-atom positions (see Section 3 below), and as a result, there are likely important δ^15^N differences among N-atom positions within molecules like arginine or ornithine.

The UC is a biochemical cycle and therefore involves chemical isotope effects, i.e., isotope selectivity during reactions. Isotope effects (denoted as α) are usually quantified using the ratio of rates or kinetic constants of the reaction of interest. They are classified into two categories: kinetic isotope effect, reflecting differences in reaction velocity (^14^*v*/^15^*v*), or thermodynamic isotope effects, reflecting differences in equilibrium constants (^14^*K*/^15^*K*). For enzymatic reactions with a Michaelian mechanism, the mathematical expression of velocity with maximal activity (*V*) and Michaelis constant (*K*) is such that the kinetic isotope effect α simplifies to the ratio of catalytic efficiencies (*V*/*K*):

α=V/K14V/K15

Bonds involving heavy isotopes (e.g., ^13^C–H, ^15^N–H, ^34^S–C) are associated with lower zero-point energy and, as a result, are generally stronger and require more energy to break. Therefore, in most reactions that break a covalent bond, reaction selectivity favours ^14^N, and thus the kinetic isotope effect is larger than unity. That said, in the case of N, it turns out that many reactions either involve rapid equilibrium during the mechanism or reversible formation of a quadrivalent ion (e.g., R–NH_2_^+^–R′), and this is associated with a thermodynamic isotope effect. It is thus not unusual to have isotope fractionations against ^14^N during some enzymatic reactions where N-atoms are involved in catalysis.

Numerically, the use of isotope effects is tedious because they are computed as ratios. It is more convenient to use the isotope fractionation (or discrimination), denoted as Δ. With some approximations, it is simply calculated using the δ^15^N-difference between the substrate and the product of the reaction of interest (in ‰):

$$\Delta =\delta_{\mathrm{substrate}}-\delta_{\mathrm{product}}$$

In this review, isotope fractionation is defined according to this convention. Since the sign convention for Δ may vary across the literature, we use this definition consistently throughout the manuscript. Negative Δ-values indicate ^15^N-enrichment in the product, thus a reaction that favours ^15^N. Positive Δ-values reflect ^15^N-depletion in the product, thus a reaction that discriminates against ^15^N. As will become apparent below (Section 3), there is quite a diversity in α (and thus Δ) values amongst UC enzymes, simply reflecting differences in chemical mechanisms during catalysis.

### 2.2. Overview of UC Enzymatic Reactions and Associated Alterations in Cancer Cells

#### 2.2.1. Rationale of UC Involvement

In cancer cells, ammonia is mostly produced during amino acid catabolism, via glutaminolysis, catalysed by glutaminase and/or glutamate dehydrogenase, producing glutamate and α-ketoglutarate (α-KG) and releasing ammonia (Figure 1). While α-KG fuels the TCA cycle (or is recycled back to citrate and oxaloacetate via ATP citrate lyase), ammonia can be either liberated, refixed into anabolic pathways, or channelled into the UC [19,20,21,22,23]. To sustain these metabolic demands, cancer cells secure glutamine supply and establish dynamic N exchange between subcellular compartments and the tumour microenvironment [24,25,26,27,28,29,30]. In this context, the UC is not only involved in ammonia detoxification but also in N partitioning between excretion and anabolic retention. This point is particularly relevant here because changes in ammonia handling and UC flux are expected to influence nitrogen isotope routing in cancer cells.

#### 2.2.2. Metabolic Steps of the UC

- ***CP metabolism: CPS1 and OTC***. Ammonia is condensed with bicarbonate to form CP by the mitochondrial enzyme CP synthase 1 (CPS1), representing the first committed step of the UC. CPS1 is tightly regulated by transcription factors (hepatocyte nuclear factor 3β, HNF3β), post-translational modifications mediated by sirtuin 5 (SIRT5), and allosteric activation by *N*-acetyl glutamate (NAG) [31,32,33,34] (Figure 1). Also, CP can be synthesised in the cytosol by the multifunctional CAD enzyme complex for de novo pyrimidine synthesis [35]. In cancer cells, mitochondrial CPS1 overexpression can divert excess CP toward cytosolic pyrimidine synthesis, as observed in non-small-cell lung cancer (NSCLC) with oncogenic KRAS/LKB1 alterations [36,37], colon cancer [38,39,40] and other tumours [41,42,43]. CPS1 downregulation, notably in hepatocellular carcinoma (HCC), may increase CAD activity and recycle ammonia through glutamine-dependent pyrimidine synthesis [44]. Ornithine transcarbamylase (OTC) condenses CP and ornithine into citrulline within mitochondria (Figure 1), with an expression modulated by SIRT3-mediated deacetylation [45]. Reduced OTC expression has been reported in HCC [46] and in paediatric sarcomas, brain tumours and acute lymphoblastic leukaemia [47,48], and is thought to increase CP availability while diverting ornithine towards alternative metabolic routes.
- ***Aspartate metabolism: citrin and ASS1***. Aspartate is amongst precursors used by nucleotide biosynthesis and is thus essential for tumour cell proliferation under both normoxic and hypoxic conditions [5,49,50,51,52,53]. Cancer cells rely heavily on endogenous production of aspartate via glutaminolysis and transamination of oxaloacetate by aspartate transaminase [54]. Mitochondrial chemistry also impacts aspartate availability via oxaloacetate provision. Thus, defects in respiration (mitochondrial electron chain) inhibit proliferation unless alternative NAD^+^-regenerating pathways compensate [49,51,52]. Cancer cells with a deficiency in succinate dehydrogenase (SDH, complex II of the mitochondrial electron chain) rely on pyruvate carboxylase to generate oxaloacetate and thus sustain aspartate production [55]. Citrin, a mitochondrial aspartate–glutamate carrier, is amplified in HCC [56], breast cancer and oesophageal cancer [57], and correlates with tumour aggressiveness in colorectal cancer [5,58]. By increasing cytosolic aspartate availability, citrin upregulation supports pyrimidine synthesis, particularly in tumours with reduced expression of ASS1 [5]. In fact, ASS1 catalyses the ATP-dependent conversion of citrulline + aspartate into argininosuccinate (Figure 1). The abundance of this enzyme is controlled by oncogenes (p53, SP4, MYC, and HIF1α) and circadian regulators (DEC1, CLOCK and BMAL1) [59,60,61,62]. The loss or silencing of the gene encoding for ASS1 induces arginine auxotrophy while overexpression in certain tumours promotes proliferation and nitric oxide (NO) production via NO synthase, contributing to tumour growth and aggressiveness [63].
- ***Arginine, fumarate and ornithine utilisation***. ASL cleaves argininosuccinate into arginine + fumarate, linking UC flux to the TCA cycle (Figure 1). In tumours with elevated ASS1 expression, ASL-driven fumarate production supports proliferation and can compensate for pharmacological ASS1 inhibition [64]. Conversely, reverse ASL activity contributes to fumarate detoxification in fumarase-deficient cancer cells, with arginine auxotrophy as a side effect [65,66]. ASL overexpression correlates with poor survival in several cancers [67,68,69] and promotes NO-dependent signalling pathways, while tumour genetic background and microenvironment also modulate the impact on tumour progression [70,71,72,73,74,75,76,77]. Arginine is further metabolised by arginase (with two isoforms, ARG1 and ARG2) into ornithine + urea, feeding polyamine synthesis, NO production and mTORC1 activation [78]. Within tumour cells, mitochondrial transporters of ornithine (ORNT1 and ORNT2) further influence N partitioning and the distribution of UC intermediates. In addition, subcellular compartmentalisation likely adds another level of complexity to isotopic routing. CPS1 generates CP in mitochondria, whereas CAD synthesises it in the cytosol, and carriers such as citrin further connect mitochondrial and cytosolic N metabolism. Therefore, differences in pool sizes, transport kinetics, and isotopic equilibration between compartments may modulate the net whole cell δ^15^N signal. Beyond these tumour cell autonomous effects, the tumour microenvironment can also reshape N handling. For example, ARG1-expressing macrophages promote tumour growth and immune suppression [79,80]. Although these processes are tightly interconnected in vivo, it is useful to distinguish tumour cell intrinsic UC rewiring from microenvironmental or systemic N handling, since the resulting isotopic signal may integrate contributions from both.

## 3. Isotope Effects in UC and Utilisation of UC Intermediates

**Figure 2:**
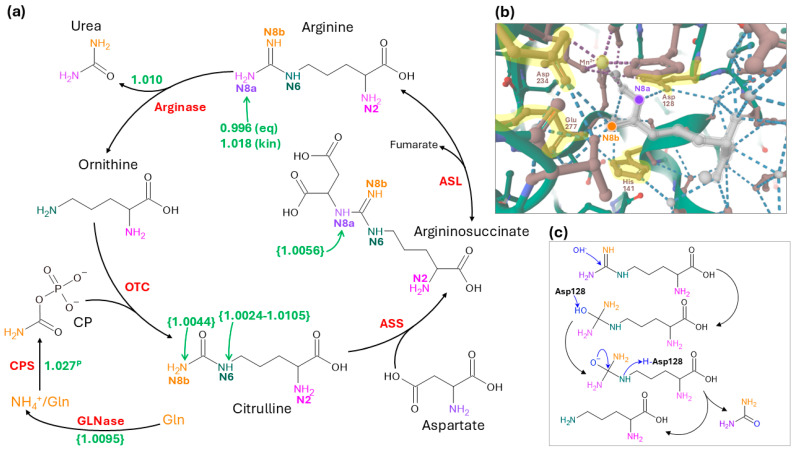
Chemistry of the urea cycle, showing key isotope effects and N-atom positions. Isotope effects in urea cycle reactions are summarised in panel (**a**), while panels (**b**,**c**) provide a structural and mechanistic focus on arginase, used here as an illustrative example of how enzyme chemistry can generate position-specific isotope effects. In (**a**), positional isotope effects are shown in green (see main text for details and enzyme abbreviations). This figure does not show isotope effects upstream of aspartate (transamination). In (**b**), the human arginase active site is shown with the bound arginine analogue *N*-hydroxyarginine. Arginine is shown in grey, with guanidium N atoms coloured in orange and purple. Important amino acids for catalysis are highlighted in yellow: Asp 234, Glu 277 and His 141 (coordination of arginine) and Asp128 (catalysis by proton relay). The 3D structure is from the Protein Data Bank, under reference number 3KV2. Coordination and hydrogen bonds are shown as dashed lines. In (**c**), a simplified formal representation of arginase catalysis is shown. For readability, only Asp128 is represented explicitly, due to its key role in proton relay from the hydroxyl group to N6. The same colours as in panel (**a**) have been used. Isotope effects that are estimated from analogous reactions rather than measured for the specific reaction of interest are shown in brackets, and the isotope effect obtained using the prokaryotic enzyme is shown with a superscript “P” after the value (e.g., 1027^P^ in panel (**a**), near CPS).

The net biochemical effect of the UC on the isotope composition of cellular material depends on three factors: (i) the isotope composition of source N feeding the UC, (ii) isotope effects (^14^*k*/^15^*k*) associated with reactions of the UC, and (iii) the isotope composition of metabolites that are abstracted from the UC (such as ornithine to produce putrescine) or excreted (such as urea). In the description below, we recall EC numbers, so that the reader can search for more chemical details on the catalytic reaction, using a query on databases such as Brenda (www.brenda-enzymes.org). N-atoms are mentioned with their number described in Figure 2. Also, note that not all isotope effects have been measured on the human enzymes, and some of them are inferred from the catalytic mechanism or from analogous enzymes. Although this represents a limitation in our current interpretation of isotope fractionations in UC and their extension to tumour biology, it still provides a useful basis to identify steps that are likely isotopically important.

The isotope composition of source N entering the cycle is primarily that of CP, the N-atom of which comes from ammonium (NH_4_^+^) or glutamine via CPS1, a glutamine- or ammonium-dependent enzyme (EC 6.3.4.16, EC 6.3.5.5). This N-atom ends up in position **N8b** of citrulline. However, as mentioned above, the N-source is ultimately glutamine since cancer cells (in particular in breast cancer) express glutaminase (glutamine → glutamate + NH_4_^+^, EC 3.5.2.1) to a high level [81]. Glutaminase, probed using the glutamine hydrolytic activity of asparaginase, is believed to be associated with an isotope effect of 1.0095, producing ^15^N-depleted ammonium [82]. CPS is associated with a substantial isotope effect against ^15^N, which has been estimated in the prokaryotic enzyme (*E. coli*) at 1.027 [83], meaning that CP is in principle depleted in ^15^N by 27‰ compared to source glutamine (amide N atom). Of course, this isotope effect might not apply directly in the cell since glutamine represents a branching point in metabolism and can be used by other metabolic pathways rather than being only committed to the UC. In addition, glutamine can be reformed from ammonium by glutamine synthase (EC 6.3.1.2). This enzyme is associated with a ^14^N/^15^N isotope effect that is quite variable depending on organisms and isoforms considered [84,85,86]. In summary, the δ^15^N value of CP is expected to depend on the balance between glutamine production and degradation and the metabolic commitment of glutamine, and may, in some cases, reflect the δ^15^N value of amide N in glutamine if the metabolic commitment to the UC is very high.

The other source of N to the UC is the amino N-atom of aspartate (which forms the **N8a** atom of argininosuccinate), via the action of argininosuccinate synthase (ASS, EC 6.3.4.5). The isotope composition of aspartate is determined by nutrition (aspartate being an important amino acid found in serum and tissues) and the isotope effect of aspartate amino transferase (EC 2.6.1.1) of 1.0056 during aspartate transamination to (or from) glutamate [87]. As a result, aspartate should be, in principle, slightly enriched in ^15^N compared to glutamate. However, to our knowledge, the isotope effect associated with ASS is unknown. A comparable reaction involving aspartate with a nucleophilic attack of a N atom on a C-atom is dihydroorotase (EC 3.5.2.3), which is associated with an isotope effect of 1.0056 during catalysis of dihydroorotate production [88]. If also applicable to ASS, this effect may thus compensate for the natural ^15^N-enrichment in the amino N-atom of aspartate and thus should not lead to the production of ^15^N-depleted **N8a** atom in argininosuccinate.

The enzyme that catalyses the entry of N from CP into the UC is ornithine carbamoyl transferase (EC 2.1.3.3). The ^15^N/^14^N isotope effect of this enzyme is currently unknown. A very similar enzyme is aspartate transcarbamoylase (EC 2.1.3.2), which is associated with an isotope effect of 1.0044 (N-atom of carbamylated substrate) and 1.0024–1.0105 (N-atom from CP) [89,90]. Therefore, under the assumption that the two enzymes are effectively similar, the entry of N into the UC probably generates naturally ^15^N-depleted citrulline on both **N6** and **N8b** atoms. Argininosuccinate lyase (EC 4.3.2.1) cleaves argininosuccinate into fumarate and arginine, with an isotope effect at **N8a** of 0.996 at equilibrium (favours ^15^N) or 1.018 during unidirectional arginine production (favours ^14^N) [91]. In vivo, it is rather unlikely that the equilibrium can be reached since the *K*_m_ for arginine and fumarate is very high (3 and 5 mM), i.e., several fold higher than that of argininosuccinate, so argininosuccinate cleavage is clearly favoured. Therefore, this enzyme contributes to depleting **N8a** in ^15^N in arginine.

The key enzyme of the UC is arginase (EC 3.5.3.1), which cleaves arginine into urea and ornithine. N-atoms in urea come from **N8a** and **N8b** of arginine, while ornithine inherits **N2** and **N6** of arginine. The reaction cleaves the C-N bond between **C6** and **N6** atoms. Arginase catalysis discriminates against ^15^N at **N8** (**a** and **b** taken together) by 10‰ (i.e., kinetic isotope effect of 1.010), while surprisingly, no isotope effect is seen at **N6**, although there is a substantial ^12^C/^13^C isotope effect of 1.015 [92]. This difference between ^13^C and ^15^N primary isotope effects likely comes from the fact that during catalysis, (i) the OH^−^ attack at **C6** produces a tetrahedral intermediate from a trigonal geometry in the guanidium group of arginine, while (ii) the geometry at **N6** changes minimally due to concerted C–N bond cleavage and N–H formation, i.e., N protonation [93,94]. Interestingly, the two N atoms of the guanidium group (**N8a** and **N8b**) do not encounter the same chemical events during the reaction: while the N atom of one of the C-NH(2) groups, is bonded to Glu 277, the N atom of the other group is closer to the metal ion in the active site and transiently forms part of a tetrahedral intermediate [93,94,95]. Note that the differentiation between **N8a** and **N8b** in the substrate (arginine) is not fixed since the two N atoms of the guanidium groups can interconvert easily (=NH ↔ –NH_2_). But in principle, the stereochemical mechanism and the structure of the active site of arginase might lead to a non-symmetrical isotope composition in urea and arginine, with distinct δ^15^N values of the two N-atoms of the guanidium. Also, an important consequence of the action of arginase is the production of ^15^N-depleted urea, due to its kinetic isotope effect. Since urea then leaves the cycle and is excreted, this contributes to ^15^N depletion in the extracellular medium and ^15^N enrichment in cellular metabolites.

Nevertheless, UC intermediates can also be used by other pathways and thus may impact the isotope composition of cells accordingly. First, amino acids such as arginine and aspartate can be used to synthesise proteins, and this fractionates against ^15^N due to the kinetic isotope effect (1.009) of ribosomal peptidyl transferase activity [96]. Second, excreted urea can be cleaved by prokaryotic urease (EC 3.5.1.5) in the gastrointestinal tract, producing ammonia that can be used, for example, by CPS. Urea-derived ammonia is slightly ^15^N-depleted since urease is associated with a small isotope effect of less than 1.002 [97,98,99]. The contribution of this process to amino acid synthesis (including arginine) is, however, believed to be very small [100]. Third, polyamines can be produced from the UC, primarily via the decarboxylation of ornithine to putrescine by ornithine decarboxylase (EC 4.1.1.17). In a similar reaction catalysed by glutamate decarboxylase, there is an inverse isotope effect of 0.9855 so that evolved γ-aminobutyrate is ^15^N-enriched [101]. On the other hand, amine oxidases (including diamine oxidase, EC 1.4.3.22, which degrades putrescine) fractionate against ^14^N, with an isotope effect within 0.991–0.997 [102,103,104,105]. As such, the δ^15^N value of polyamines, which are either incorporated into cellular metabolism or excreted, basically depends on the balance between production and degradation. Also, it is important to remember that the isotope effect associated with deprotonation of –NH_3_^+^ groups to –NH_2_ is highly significant, about 1.022 in amino acid NH_2_ groups [104] and 1.019 for free ammonia [106]. For example, polyamines are transported in their positively charged (thus ^15^N-enriched) form, and thus, polyamine excretion abstracts ^15^N-enriched material (with respect to, e.g., ornithine, which has a lower pK_a_ (≈9) at **N2** than putrescine pK_a_, ≈10). The same applies to ammonium, which can deprotonate and leave to the extracellular medium. In principle, we anticipate this effect to be enhanced in cancerous tissues since extracellular pH is lower due to lactic acidosis (caused by the Warburg effect under hypoxic conditions).

In summary, the UC is associated with many isotope effects (Figure 2), and as a result, it produces ^15^N-depleted arginine and urea, because both N entry into the cycle (glutaminase, CPS) and urea production (arginase) fractionate against ^15^N. However, the overall impact of the UC on cellular δ^15^N also depends on isotope effects in ancillary metabolism, such as polyamine metabolism and excretion, and ammonium exchange. Possible effects of the UC and N excretion on the ^15^N depletion reported in cancer cells are discussed just below in Section 4.

## 4. Consequences for Cancer Cells: Recurrent ^15^N-Depletion

**Figure 3:**
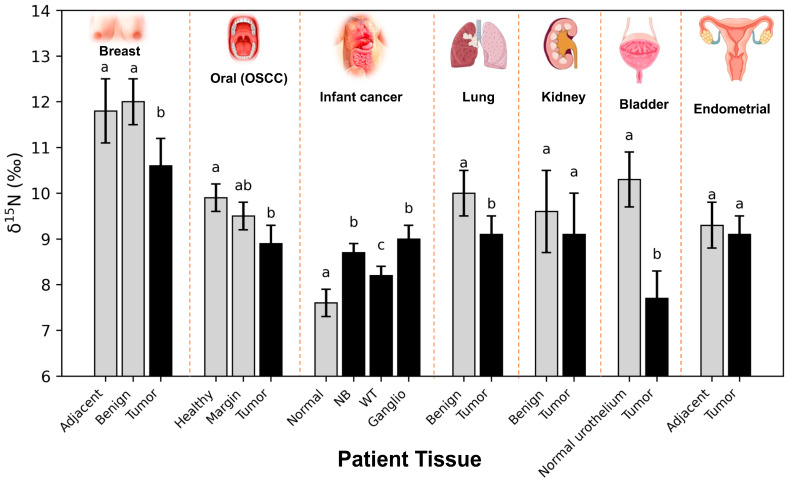
**Qualitative overview of reported δ^15^N values in cancer.** The studies summarised here include breast cancer tissues, oral squamous cell carcinomas (OSCC) [107] and patients with paediatric cancer [12,13] and adjacent non-cancerous tissue when available. Comparators differ between studies. Accordingly, this figure should be interpreted as a qualitative synthesis rather than as a directly comparable quantitative summary. Note that δ^15^N values in OSCC (oral squamous cell carcinoma) tumour tissues slightly differ from those in tissues from the margin and distant oral mucosa (OSCC healthy) [107]. In babies or children, δ^15^N values from ganglioneuroma (ganglio, benign tumours), neuroblastoma (NB), and nephroblastoma (Wilms’ tumours, WT, which are malignant) are compared with normal kidney cortex tissue (Normal), used as a control [12,13,16]. Changes in δ^15^N are also observed in various cancer types, including bladder and lung cancers [14,16] and endometrial cancer [15]. Letters above the bars indicate statistical groupings as reported in the original studies; within each individual study/comparison, bars with different letters are significantly different, whereas bars sharing at least one letter are not significantly different.

Figure 3 provides a qualitative overview of published δ^15^N patterns across tumour types and experimental systems. Because the available studies rely on heterogeneous biological materials and comparators (e.g., adjacent tissue, distant mucosa, benign tumours, paediatric control tissue, or non-cancerous cultured cells), these datasets should not be interpreted as directly quantitatively comparable across studies. In addition, most published isotopic studies do not include parallel characterisation of key UC enzymes or matched metabolic flux data. As a result, comparisons across tumour types remain difficult to interpret at the mechanistic level, and the framework proposed here should be viewed as a general explanatory model rather than as a tumour-type-specific mechanism already validated across cancers. To date, stable isotope analyses performed in tumour tissues and cultured cancer cell lines have frequently reported ^15^N depletion and, under some circumstances (Figure 3), an enrichment in ^13^C relative to healthy counterparts (adjacent tissue or non-cancerous cultured cells). These isotopic differences have been observed across different experimental systems, including breast cancer biopsies and cultured breast cancer cells, and across various cancer types, including human breast cancer [11,16], oral cancer [107,108], bladder and lung cancers [14,16], endometrial cancer [15] (Figure 3), and mouse brain and head and neck cancers [109]. In addition, reduced ^15^N content has been reported in several human breast [11,16], colorectal [17] and prostate [110] cancer cell lines, indicating that these signatures reflect intrinsic features of cancer metabolism rather than artefacts of nutrition or sample preparation. This raises the question of the metabolic origin of ^15^N-depletion, and the UC is believed to play a role here.

When observed, ^15^N depletion in cancer tissues likely reflects the balance between N source (glutamine) and N excretion (via, e.g., urea, arginine or ammonium), favouring the preferential capture of ^14^N. In practice, it relates to N fluxes associated with glutamine catabolism (influx) and nitrogenous compounds liberated from UC reactions (efflux). As mentioned above, glutaminolysis is the major N entry point in cancer cells, involving glutaminase-mediated deamidation of glutamine and the release of ammonia. Interestingly, glutamine itself is naturally ^15^N-depleted relative to bulk dietary N, mainly due to strong ^15^N depletion of its amide N (δ^15^N ≈ −6.5‰ on average), while the amine N remains close to bulk values, as shown by Position Specific Isotope Analysis [111]. Both glutaminase activity and UC reactions are associated with kinetic isotope effects that discriminate against ^15^N, so that urea and arginine are naturally ^15^N-depleted (see Section 3). This process probably explains why glutamine-dependent cancer cell metabolism leads to the accumulation of ^15^N-depleted cellular N pools.

This ^15^N-depleting effect of metabolism is further exaggerated by the fact that the efflux of ammonium, arginine and urea into the extracellular medium is not quantitative and does not balance N influx. In addition, urea released by tumour cells should not necessarily be viewed as irreversibly lost from the local system, since local retention within the tumour microenvironment or partial recycling, including via microbial urease activity, could modulate N fluxes and thus influence the resulting isotopic signature. Instead, a significant fraction of N is retained intracellularly, leading to a relatively high elemental N content (%N) in tumours [11]. Retained N is recycled into downstream anabolic pathways, including polyamine synthesis and β-alanine metabolism (Figure 1). Increased polyamine metabolism, a hallmark of many cancers, has been amply documented and represents an efficient sink for recycled N [112,113,114,115]. Also, the proportion of nitrogenous compounds in tumours (proteins and nucleic acids) is higher than in healthy tissues at the expense of lipid content (this effect is evident in the case of breast cancer, where %N can increase by up to ~200% [11]). This general effect is such that a depletion of ^15^N in bulk organic matter (and not only specific, isolated metabolites) is visible. It is also worth noting that the tumour microenvironment is acidic, and thus, all molecules that are released by cells in the extracellular medium are in their protonated state (–NH_3_^+^), which is enriched in ^15^N compared to the deprotonated state (isotope effects in protonation are discussed in Section 3). This also likely contributes to the ^15^N-depletion of cancer cells. In summary, the isotope composition in cancer is determined by a combination of mass-balance (influx-efflux difference) phenomena, with a probable contribution of isotope effects in UC.

It is worth noting that in addition to changes in N isotopes, cancer tissues often display an enrichment in ^13^C. This ^13^C-enrichment comes from both the lower content in lipids (which are generally ^13^C-depleted) and the reorchestration of metabolism, which promotes ^13^C-enriching reactions, including UC. In effect, the ^13^C-enrichment can be explained by anaplerotic fixation of bicarbonate, which is naturally ^13^C-enriched, via pyruvate carboxylase, phosphoenolpyruvate carboxylase or CPS. That is, the ^13^C-enrichment partly relates to UC, via the action of CPS, which introduces a ^13^C-enriched C-atom into ornithine and downstream metabolites (including arginine). Therefore, the build-up of arginine and polyamine in cancer cells contributes to the observed cellular ^13^C-enrichment. Interestingly, recent isotopic analyses have shown that the lipid isotope composition (δ^13^C) is closely related to that of total organic matter (and thus is not systematically considerably ^13^C-depleted), and that variations in lipid abundance are insufficient to explain the observed ^13^C-enrichment in cancer tissues. This supports the idea that modifications in C and N primary metabolic pathways (including the enhancement of UC) are important drivers of δ^15^N of bulk cellular material, aside from broad compositional changes such as lipid content [116].

Quite critically, the isotopic pattern found in tumour biopsies and that in cultured cancer cell lines are very similar. In fact, both biological materials show a ^15^N-depletion (compared to healthy tissues and non-cancerous cultured cells) and, in most cases, a ^13^C-enrichment, despite differences in nutrient availability and microenvironment. This convergence strongly argues against a prevalent role of dietary inputs and rather highlights the central role of tumour-specific metabolism. While the origin of the ^13^C-enrichment somewhat differs between in vitro and in situ situations (i.e., with variable importance of lipid metabolism), the origin of the ^15^N-depletion seems to be conserved. In cultured cells, increased UC activity combined with incomplete excretion of ^15^N-depleted arginine drives intracellular ^15^N loss. In some tumours, arginine auxotrophy and enhanced recapture of extracellular, ^15^N-depleted arginine support polyamine synthesis and biomass production, resulting in further ^15^N-depleted cellular material. This mechanism aligns well with established features of tumour biology (recalled in the Introduction), including increased polyamine demand, differential expression of arginase, and the identification of urea- and glutamine-related pathways as metabolic biomarkers of breast cancer.

Taken as a whole, ^15^N natural abundance appears to integrate metabolic fluxes in N metabolism, such that δ^15^N differs between healthy and cancerous cells. In this sense, δ^15^N may provide a useful functional readout of metabolic state. Further, it may help to distinguish tumour grade, severity, or metabolic subtype [116], because cancer cell subtypes are often associated with subtle metabolic changes that could translate into ^15^N abundance isotopic differences, as has indeed been shown between cultured cancer cell lines [11,17,109,110]. Given the metabolic heterogeneity of breast cancer subtypes, isotope-based approaches could thus complement existing molecular classifications by providing a functional, flux-integrated probe of tumour metabolism. However, the evidence summarised here is based predominantly on tumour tissues and cultured cancer cell lines, and not yet on validated biofluid-based clinical studies. From a translational perspective, isotopic measurements would be more useful if applied to accessible biofluids rather than bulk material (from, e.g., biopsies or exeresis samples). In this context, plasma metabolites, such as arginine, represent promising candidates for future investigations. If tumour activity alters the circulating isotopic pool to a measurable extent, then compound-specific δ^15^N measurements could become informative for cancer phenotyping or monitoring, but this remains to be demonstrated in prospective clinical studies.

## 5. Perspectives

While this review mainly focuses on ^14^N/^15^N isotope effects associated with UC metabolism, similar principles apply to other isotopes, the natural abundance of which may vary with metabolic fluxes and pathway modifications. To date, however, isotopic variations in elements other than C and N have been less explored in cancer tissues. For example, some studies have examined the natural ^34^S abundance in cancer. No significant difference in ^34^S was reported between bladder tumours and normal bladder tissue [9], whereas higher δ^34^S values were observed in most cancerous endometrium samples compared to distant non-cancerous tissue [11]. In addition, lower ^34^S abundance was reported in serum and erythrocytes of patients with hepatocellular carcinoma [117]. These observations suggest that sulphur isotopes may capture alterations in S-containing amino acid metabolism and redox balance in cancer, warranting further investigation. Natural variations in hydrogen isotopes (^2^H abundance) have not yet been systematically measured in tumour tissues. Recent studies provide preliminary evidence for metabolic control of ^2^H distributions. A depletion in ^2^H was observed in fatty acids from *Saccharomyces cerevisiae* undergoing fermentation compared to aerobic respiration, and a similar ^2^H depletion was reported in a liver cancer cell line relative to normal primary hepatocytes [118]. These findings perhaps indicate a link between ^2^H abundance and the enhancement of glycolysis (as opposed to aerobic metabolism) and thus may reflect a typical feature of cancer cell metabolism. For other isotopes, including metals, interesting results have been obtained with copper (Cu), zinc (Zn) and potassium (K). Higher natural ^65^Cu abundance has been reported in tumour tissues from liver [117], ovary [119], oral cavity [120], and thyroid [121] cancers compared to matched plasma samples. By contrast, while a depletion in ^66^Zn was observed in breast tumour tissue relative to non-tumour tissue, this difference was not statistically significant in paired comparisons with adjacent tissue [122], and no significant ^66^Zn variations were reported in oral cancer [120]. Early studies from the mid-20th century also reported natural ^41^K depletion in human and rodent tumours [123,124,125], and such observations would deserve re-examination using modern analytical techniques [126]. Notably, ^41^K depletion was also observed in muscles of tumour-bearing rodents [125], consistent with more recent reports of increased ^41^K excretion in the urine of patients with pancreatic cancer [127]. Given the central role of potassium as the most abundant intracellular cation and the involvement of potassium channels in cancer metabolism [128], with an expected preference for ^39^K [129], potassium isotope fractionation represents a promising but largely unexplored avenue.

Altogether, a multiple isotopic approach may provide complementary and mechanistically informative readouts for cancer metabolic phenotyping, and could eventually help characterise cancer tissues and cells. Of course, it is presently challenging to carry out multiple isotope measurements (in particular, for metals, which require specific and expensive mass spectrometers), which represents a hurdle in the current utilisation of isotopes. By contrast, measurements of C and N isotopes can be implemented routinely. As discussed in Section 4, coupling δ^15^N and δ^13^C values should provide complementary and mechanistically linked information about tumour metabolism, in particular, the enhancement of the UC. In other words, natural isotope abundances are reflective of cancer metabolism, giving some new opportunities for metabolic phenotyping, biomarker discovery, and cross-cancer comparisons. Future studies are warranted to address this aspect more deeply with intramolecular, site-specific ^15^N analyses (for example, of arginine, which contains four N-atoms) since changes in individual UC reactions should modify isotopic differences between N-atom positions, opening avenues for a high-tech, sensitive probe of cancer cell metabolism.

## Data Availability

No new data were created in this study. Data sharing is not applicable.
